# Bridging gender health gaps through digital health interventions: integrating digital health literacy

**DOI:** 10.4069/whn.2025.04.04

**Published:** 2025-05-20

**Authors:** Seul Ki Choi

**Affiliations:** Graduate School of Urban Public Health, University of Seoul, Seoul, Korea

## Introduction

Advances in digital technology have led to the development of various digital health tools, from online health magazines to real‑time remote patient monitoring via mobile apps and telemedicine. These innovations support health promotion, disease prevention and management, and patient empowerment. Moreover, recent breakthroughs in artificial intelligence (AI) across multiple healthcare domains promise even better outcomes. Thus, digital interventions have emerged as powerful means to improve health and broaden access to care [1,2].

Gender inequities in health reflect systematic differences in outcomes and access based on gender. Although medical technologies and healthcare services have grown substantially over the past century, significant disparities persist. Women generally live longer than men but spend fewer years in good health and experience higher rates of physical and mental illness and disability [3]. Furthermore, intersecting vulnerabilities, such as socioeconomic status, race, and geographic location, compound power imbalances, resource gaps, and social norms that exacerbate these inequities [4]. Digital health interventions leveraging various tools thus hold promise for addressing gender‑based health disparities [2].

Digital health technologies offer a wide range of solutions to address women’s unique health needs, including menstrual health, fertility, pregnancy, childbirth, postpartum care, and sex-specific diseases such as breast cancer and cervical cancer [5]. These tools help women manage their health, access care, and stay informed, especially in areas where traditional healthcare is hard to reach. In a recent report, the World Health Organization recognized the potential of digital health technologies to improve women’s health [2], noting that digital literacy can empower women to overcome traditional barriers and narrow gender‑based health gaps [2]. However, many women, particularly those from marginalized groups, are excluded from digital health technologies due to multiple intersecting factors including limited access to technology and lower digital health literacy (DHL). These barriers may hinder the effectiveness of digital health technologies in closing the gender health gap [2]. Therefore, well-designed digital health interventions that improve women’s capacity to effectively use digital health technology can benefit women’s health, whereas poorly designed or reckless digital health interventions risk widening existing gender health disparities.

This paper treats DHL as a critical design element for equitable digital health interventions that advance gender equity in health. First, it outlines the current digital gender divides and the role of DHL in digital health initiatives. Then, it proposes strategies to enhance intervention effectiveness by integrating DHL considerations and promoting gender‑equitable outcomes.

## Digital gender divides

Despite rapid technological advances, gender gaps in both access to and skills for using digital technology persist worldwide. In 2023, 70% of men globally had internet access compared with 65% of women—a disparity most pronounced in the least developed countries. In high‑income nations, usage rates are nearly equal (94% of men vs. 93% of women), but in low‑income countries, only 34% of men and 20% of women go online [6]. Although smartphone and mobile internet adoption in low‑ and middle‑income countries has improved, women still lag behind men in device ownership and mobile data use [7,8].

Importantly, disparities in digital technology usage exceed those in mere access. Having connectivity does not guarantee sufficient digital skills: women are less likely than men to possess adequate digital literacy [8,9]. This skills gap is widest in low‑income countries [9] and persists even in high‑income nations where access is comparable [10,11]. Moreover, proficiency declines with age: a German study found no literacy gap among children, yet one appeared in upper secondary education and endured into adulthood [12]. Nevertheless, younger cohorts also face divides, driven in part by cultural and structural barriers to education that contribute to broader health inequities [4,9].

## Digital health literacy

Digital literacy has become an essential capacity in our increasingly digital society. The United Nations Educational, Scientific and Cultural Organization defines digital literacy as “the confident and critical use of a full range of digital technologies for information, communication and basic problem-solving in all aspects of life” [13]. This concept encompasses both technical and cognitive skills, enabling individuals to effectively find, evaluate, create, and communicate information using digital platforms [14].

DHL, also known as electronic health (eHealth) literacy, is defined as “the ability to seek, find, understand, and appraise health information from electronic sources and apply the knowledge gained to addressing or solving a health problem” [15]. Although “digital health” now encompasses eHealth, mobile health (mHealth), internet of things (IoT), big data, AI, robotics, and more [1,16], the terms “eHealth literacy” and “DHL” denote the same core competencies for engaging with electronic health technologies [17]. They are therefore used interchangeably.

Several frameworks have been developed to conceptualize DHL. Norman and Skinner [15] describe it as comprising analytic literacies (traditional, media, and information) and context‑specific literacies (computer, health, and scientific)—six literacies essential for digital health engagement. Norgaard et al. [18] proposed seven core competencies of DHL at the individual level (ability to process information and engage in one’s own health), system level (access to systems that work and digital services that suit individual needs), and interaction between the two levels (ability to engage actively with digital services, feeling safe and in control, and motivation to engage with digital services). The transactional model of eHealth Literacy operationalizes these competencies through four literacies: functional, communicative, critical, and translational [19]. Ban et al. [17], based on a systematic review of 32 studies, defined DHL as “the ability to convert knowledge into practical actions by processing, communicating and utilizing health information from digital platforms, while continually regulating the knowledge translation process aligned with one’s health goals.” They identified functional literacy, experience with one’s health, and access to technology as antecedents of DHL [17]. Although each framework blends digital and health literacies, DHL extends beyond their sum: it requires proficiency in navigating digital interfaces, assessing online content credibility, and employing digital health tools [15,17,18]. Notably, the additional skills demanded by DHL may not be present even among individuals with high health literacy [20,21].

## Digital health literacy and digital health interventions

Digital health interventions require participants to use digital technologies to find information or manage their health. In other words, digital health interventions assume participants have a certain level of DHL—unless the intervention specifically aims to improve it. Yet participants’ DHL can vary widely, and not everyone meets the required threshold. Interventions that require extensive interaction—such as searching for or interpreting health information via digital tools—demand higher DHL. In contrast, those focusing on simple behavior tracking via digital devices require less interaction and may suit individuals with lower DHL. However, even these interventions still require participants to understand and apply digital health data (for example, sensor readings) to their health management. Consequently, intervention effectiveness can differ based on participants’ DHL, and neglecting DHL in design and implementation may lead to negative consequences.

### Participant recruitment

Interventions that overlook DHL risk failing to recruit a diverse population. Many studies set inclusion criteria—often implicitly—based on participants’ digital skills and ability to read and comprehend health information. As a result, individuals with limited DHL may be excluded. Even when offline recruitment or purposive sampling is used, the requirement to use digital devices may discourage those with low DHL, who may be unaware of digital health services or view them as irrelevant.

### Adoption and engagement

Both the extent of required digital interaction and the complexity of technologies can influence engagement among people with varying DHL. Women with low DHL, for example, may find digital health platforms difficult to navigate, leading to lower participation. In an asthma management study using a mobile app, participants with higher DHL showed greater engagement [20]. Low DHL is often linked to low self‑efficacy in using technology, and women frequently report less confidence in their digital skills compared to men [9]. When participants doubt their ability to use digital tools, they may engage less, reducing the effectiveness of the intervention.

Moreover, complex digital health technologies often require significant time and effort. Cultural barriers and traditional gender roles—such as caregiving responsibilities—may limit women’s availability for time‑consuming digital tasks. For women with low DHL, these demands can feel burdensome, potentially leading to reduced engagement or dropout [22].

### Misinformation and health risks

When digital health interventions require participants to obtain health information online, individuals with low DHL may struggle to identify credible sources among vast content. They may misinterpret information, leading to harmful self‑medication or neglect of necessary care.

Interventions that incorporate social networking services (SNS) and AI-generated content may pose additional risks for participants with low DHL. Users with low DHL are more susceptible to unverified SNS information and algorithmic biases [23], and they may fail to recognize AI‑generated errors, increasing exposure to misleading health content.

## Integrating digital health literacy into digital health interventions

Because low DHL often overlaps with poor health status—and women are disproportionately represented among those with low DHL—interventions that serve only digitally literate individuals risk perpetuating or widening gender health disparities. Thus, while expanding access to digital technologies is important, digital health interventions must also address DHL to ensure equitable benefits and harness digital health’s potential to reduce gender health disparities [2]. The following strategies can help integrate DHL effectively.

### Increasing awareness of digital health literacy among intervention personnel

To develop digital health interventions that effectively address DHL, the first step is to raise awareness among all intervention personnel—developers, implementers, and evaluators. They must understand what DHL encompasses and recognize how participants’ varying DHL levels can influence critical components of an intervention, including registration, adherence, engagement, and outcomes. They should also identify barriers associated with low DHL—such as challenges in navigating digital platforms, interpreting online health information, and applying it to health decisions—especially among women and other vulnerable groups.

### Co-designing for universal design and universal precautions

Designing digital health interventions that all users can engage with, regardless of their DHL level, requires both universal design and universal precautions. Universal design principles, which were originally intended to improve accessibility in the built environment [24], have been adapted for digital contexts. In digital health technology, these principles include simple and intuitive interfaces, low physical effort, flexible user settings, and tolerance for error, which together enhance accessibility and usability for everyone, including those with limited digital skills [24]. Applied effectively, universal design can make the healthcare system more inclusive and equitable [25]. However, universal design alone may not fully address the needs of individuals with limited DHL, who may still struggle to interpret the information presented.

The universal precaution principle in health literacy operates on the assumption that everyone may have limited health literacy. By communicating health information clearly and simply, this approach complements universal design. Since providers cannot always determine who understands health information—and even those with adequate literacy may struggle when ill or stressed—treating all users as if they have limited literacy ensures information is clear, actionable, and understandable, thereby reducing disparities in healthcare access and outcomes [26,27]. In digital health interventions, intervention personnel cannot reliably identify participants’ DHL without formal assessment, and even those with digital access may find it challenging to comprehend or use digital health information. Therefore, adopting universal precautions in digital interventions ensures that all participants, regardless of gender or DHL level, can understand and use the information for health decision‑making and self‑care.

Co‑design is critical for integrating universal design and universal precautions into digital health interventions [26,27]. Participants best understand their own health needs, the challenges posed by interventions, the accessibility of digital tools, and the unique barriers to their engagement. Collaborating with participants—especially those with limited DHL—in the design and testing phases enhances the accessibility, understandability, and equity of interventions across all DHL levels. For example, wMammogram, a mobile app-based intervention promoting breast cancer screening among American Indian women, involved its target group in development. Through focus groups, the team identified barriers to screening, mobile phone usage patterns, and preferred health message formats, which informed both content and reminder schedules. Additionally, a nurse health navigator provided technical support, and the research team lent mobile phones to participants. This approach yielded high engagement, satisfaction, completion rates, and improved screening outcomes compared to controls [28].

### Digital health literacy assessment

Digital health interventions should assess participants’ DHL at baseline and adjust the complexity of digital tools accordingly, providing tailored DHL education and training as needed. In other words, measuring DHL enables customization of interventions so that individuals at all DHL levels can fully engage with each component. Including DHL assessments in evaluation plans is also essential to determine whether intervention effectiveness varies by gender and DHL level, thereby assessing the impact on gender health disparities.

DHL can be measured using self‑reported instruments such as the eHealth Literacy Scale (eHEALS), the eHealth Literacy Questionnaire (eHLQ), and the Digital Health Literacy Instrument (DHLI). Although eHEALS is the earliest and most widely used measure, its 20‑year‑old design may not capture the competencies required by today’s evolving technologies [29]. Other instruments also target different domains—some focus solely on internet‑related DHL, while others are specific to mobile technologies [16]. Therefore, intervention personnel should be familiar with various DHL measures and select the one most appropriate for their intervention’s purpose and methods.

## Conclusion

As digital tools become increasingly prevalent, digital skills, access to digital services, and DHL are recognized as “super social determinants of health” because they profoundly influence all other determinants [30]. While digital health interventions have the potential to narrow gender health gaps, they can also widen them if DHL is overlooked. Considering DHL in digital health interventions is thus a key strategy for maximizing benefits and minimizing risks.

Incorporating DHL into digital health interventions requires collaboration across government agencies, private institutions, and researchers. Government initiatives are vital to ensure that advancements in digital health technology do not exclude women, especially those with low DHL [1]. National digital health strategies should include measures to expand digital infrastructure and improve DHL at the population level. Integrating DHL education into public curricula and continuing education ensures lifelong benefits for both women and men. Public health professionals also need DHL training to enhance their competencies and understand the effects of variable DHL among patients. Moreover, governments, in collaboration with stakeholders, should develop guidelines for incorporating DHL into digital health interventions. Ongoing attention and effort are necessary to ensure that varying DHL levels do not become barriers to participation and engagement, thereby allowing all individuals to reap the health benefits of digital interventions.

## References

[1] World Health Organization (2021). Global strategy on digital health 2020–2025 [Internet]. https://www.who.int/publications/i/item/9789240020924.

[2] World Health Organization Regional Office for Europe (2024). The role of digital health technologies in women’s health, empowerment and gender equality: project report [Internet]. https://www.who.int/europe/publications/i/item/WHO-EURO-2024-9293-49065-73153.

[3] Carmel S (2019). Health and well-being in late life: gender differences worldwide. Front Med (Lausanne).

[4] Morgan R, Ayiasi RM, Barman D, Buzuzi S, Ssemugabo C, Ezumah N (2018). Gendered health systems: evidence from low- and middle-income countries. Health Res Policy Syst.

[5] Lupton D, Maslen S (2019). How women use digital technologies for health: qualitative interview and focus group study. J Med Internet Res.

[6] International Telecommunication Union (ITU) (2023). Measuring digital development: facts and figures 2023 [Internet]. https://www.itu.int/itu-d/reports/statistics/facts-figures-2023/.

[7] GSM Association (GSMA) (2024). The mobile gender gap report 2024 [Internet]. https://www.gsma.com/r/wp-content/uploads/2024/05/The-Mobile-Gender-Gap-Report-2024.pdf.

[8] Sey A, Hafkin N (2019). Taking stock: data and evidence on gender equality in digital access, skills and leadership [Internet]. https://i.unu.edu/media/cs.unu.edu/attachment/4040/EQUALS-Research-Report-2019.pdf.

[9] Tyers-Chowdhury A, Binder G (2021). What we know about the gender digital divide for girls: a literature review [Internet]. https://www.unicef.org/eap/media/8311/file/What%20we%20know%20about%20the%20gender%20digital%20divide%20for%20girls:%20A%20literature%20review.pdf.

[10] European Institute for Gender Equality (c2025). Gender Equality Index: digitalisation in the world of work [Internet]. https://eige.europa.eu/gender-equality-index/thematic-focus/digitalisation/country.

[11] Perifanou MA, Economides AA (2020). Gender digital divide in Europe. Int J Bus Humanit Technol.

[12] Bachmann R, Hertweck F (2025). The gender gap in digital literacy: a cohort analysis for Germany. Appl Econ Lett.

[13] United Nations Educational, Scientific and Cultural Organization (UNESCO) (2021). SDG 4: Ensure inclusive and equitable quality education and promote lifelong learning opportunities for all [Internet]. https://tcg.uis.unesco.org/wp-content/uploads/sites/4/2021/08/Metadata-4.4.2.pdf.

[14] American Library Association Digital Literacy Taskforce (2011). What is digital literacy? [Internet]. https://alair.ala.org/server/api/core/bitstreams/6a42b84d-dbaf-4871-b2da-d60b51b21aef/content.

[15] Norman CD, Skinner HA (2006). eHealth literacy: essential skills for consumer health in a networked world. J Med Internet Res.

[16] van der Vaart R, Drossaert C (2017). Development of the digital health literacy instrument: measuring a broad spectrum of Health 1.0 and Health 2.0 skills. J Med Internet Res.

[17] Ban S, Kim Y, Seomun G (2024). Digital health literacy: a concept analysis. Digit Health.

[18] Norgaard O, Furstrand D, Klokker L, Karnoe A, Batterham R, Kayser L (2015). The e-health literacy framework: a conceptual framework for characterizing e-health users and their interaction with e-health systems. Knowl Manag E-Learn.

[19] Paige SR, Stellefson M, Krieger JL, Anderson-Lewis C, Cheong J, Stopka C (2018). Proposing a transactional model of eHealth literacy: concept analysis. J Med Internet Res.

[20] Silverstein GD, Styke SC, Kaur S, Singh A, Green S, Jariwala SP (2023). The relationship between depressive symptoms, eHealth literacy, and asthma outcomes in the context of a mobile health intervention. Psychosom Med.

[21] Monkman H, Kushniruk AW, Barnett J, Borycki EM, Greiner LE, Sheets D (2017). Are health literacy and eHealth literacy the same or different?. Stud Health Technol Inform.

[22] Veinot TC, Mitchell H, Ancker JS (2018). Good intentions are not enough: how informatics interventions can worsen inequality. J Am Med Inform Assoc.

[23] Okan O, Messer M, Levin-Zamir D, Dadaczynski K, Paakkari L, Schaeffer D (2023). Health literacy action framework for health emergencies and infodemics. Inf Serv Use.

[24] Story MF, Preiser WF, Smith KH (2010). Universal design handbook.

[25] Xie Y, Fadahunsi KP, Kelleher C, Tarn DM, Grace A, O’ Donoghue J (2025). Towards an inclusive digital health ecosystem. Bull World Health Organ.

[26] DeWalt DA, Broucksou KA, Hawk V, Brach C, Hink A, Rudd R (2011). Developing and testing the health literacy universal precautions toolkit. Nurs Outlook.

[27] U.S. Department of Health and Human Services, Office of Disease Prevention and Health Promotion (2015). Health literacy online: a guide to simplifying the user experience [Internet]. https://odphp.health.gov/healthliteracyonline/.

[28] Roh S, Lee YS, Kenyon DB, Elliott AJ, Petereit DG, Gaba A (2023). mobile web app intervention to promote breast cancer screening among American Indian women in the Northern Plains: feasibility and efficacy study. JMIR Form Res.

[29] Norman C (2011). eHealth literacy 2.0: problems and opportunities with an evolving concept. J Med Internet Res.

[30] Hanebutt R, Mohyuddin H (2023). The digital domain: a “super” social determinant of health. Prim Care.

